# Timing and Duration of Drug Exposure Affects Outcomes of a Drug-Nutrient Interaction During Ontogeny

**DOI:** 10.3390/pharmaceutics2040321

**Published:** 2010-10-14

**Authors:** Binbing Ling, Caroline Aziz, Chris Wojnarowicz, Andrew Olkowski, Jane Alcorn

**Affiliations:** 1 College of Pharmacy and Nutrition, University of Saskatchewan, 110 Science Place, Saskatoon, S7N5C9, Canada; 2 Toxicology Centre, University of Saskatchewan, 44 Campus Drive, Saskatoon, SK, S7N 5B3, Canada; 3 Department of Veterinary Pathology, Prairie Diagnostic Services, 52 Campus Drive, University of Saskatchewan, Saskatoon, SK, S7N 5B4, Canada; 4 Department of Animal and Poultry Science, University of Saskatchewan, 51 Campus Drive Saskatoon, SK, S7N 5A8, Canada

**Keywords:** drug-nutrient interaction, ontogeny, L-carnitine, cefepime, windows of susceptibility

## Abstract

Significant drug-nutrient interactions are possible when drugs and nutrients share the same absorption and disposition mechanisms. During postnatal development, the outcomes of drug-nutrient interactions may change with postnatal age since these processes undergo ontogenesis through the postnatal period. Our study investigated the dependence of a significant drug-nutrient interaction (cefepime-carnitine) on the timing and duration of drug exposure relative to postnatal age. Rat pups were administered cefepime (5 mg/kg) twice daily subcutaneously according to different dosing schedules (postnatal day 1-4, 1-8, 8-11, 8-20, or 1-20). Cefepime significantly reduced serum and heart L-carnitine levels in postnatal day 1-4, 1-8 and 8-11 groups and caused severe degenerative changes in ventricular myocardium in these groups. Cefepime also altered the ontogeny of several key L-carnitine homeostasis pathways. The qualitative and quantitative changes in levels of hepatic γ-butyrobetaine hydroxylase mRNA and activity, hepatic trimethyllysine hydroxlase mRNA, intestinal organic cation/carnitine transporter (Octn) mRNA, and renal Octn2 mRNA depended on when during postnatal development the cefepime exposure occurred and duration of exposure. Despite lower levels of heart L-carnitine in earlier postnatal groups, levels of carnitine palmitoyltransferase mRNA and activity, heart Octn2 mRNA and ATP levels in all treatment groups remained unchanged with cefepime exposure. However, changes in other high energy phosphate substrates were noted and reductions in the phosphocreatine/ATP ratio were found in rat pups with normal serum L-carnitine levels. In summary, our data suggest a significant drug-nutrient transport interaction in developing neonates, the nature of which depends on the timing and duration of exposure relative to postnatal age.

## 1. Introduction

Early postnatal life represents a period of significant susceptibility to nutritional alterations that may affect maturing biochemical and physiological processes with long-term consequences on individual health [1,2]. Developing individuals can adapt to nutritional changes to favor survival [3,4] and these adaptations can be subtle or obvious and transient or permanent depending on factors such as the stage of physiological development, duration of nutritional alteration and whether the alteration occurs during a critical window of susceptibility [5,6,7]. When neonates become ill, pharmacological interventions are often necessary to reduce the burden of disease-associated morbidity and mortality. Despite a general acknowledgement that drug use may impact normal development of the physiological system [8], limited research has examined the consequences of pharmacological interventions on the developing neonate, particularly when drugs influence nutrient availability in the body [9]. 

Both nutrient homeostasis pathways and processes governing drug pharmacokinetics in neonates may undergo significant ontogenesis in the postnatal period [10]. When a drug and nutrient share the same absorption and/or disposition mechanism, a significant drug-nutrient interaction during postnatal development is possible and this interaction could affect the ontogeny of these pathways. Such effects may be transient with the normal genetic programming restored upon removal of the drug exposure, or permanent changes may occur when drug exposure occurs during a critical window of susceptibility [11,12]. Whether such changes are possible is mostly speculative as the majority of investigations into drug-nutrient interactions examine the influence of nutrients on drug pharmacokinetics and/or pharmacodynamics typically in adult populations [13,14,15,16,17]. Fewer studies have evaluated the influence of drugs on nutrient homeostasis [9,18]. 

Transport of substrates across epithelial barriers and into and out of cell and cellular organelles via a transport protein is a process (either for absorption into the body or for distribution or elimination from the body) often shared by both a drug and a nutrient [19]. Transport systems are particularly important to nutrients to make them available to the physiological system and maintain nutrient homeostasis. When a transporter mediates both nutrient and drug transport across membranes, drug therapy in the neonate could result in potential reversible interactions between a drug and a nutrient that may alter nutrient absorption and disposition in the developing individual (and *vice versa*). At present, very little data is available on the potential risk of putative drug-nutrient interactions at transport systems that cause nutritional alterations in the neonate. Furthermore, the significance of a putative drug-nutrient transporter interaction in a neonatal patient could vary depending on the magnitude and duration of a drug exposure and the timing of that exposure during postnatal development. Such factors may lead to different adaptive strategies to optimize nutrient homeostasis in a neonate and may consequently lead to significant changes in neonatal development especially when the nutrient affected is critical for neonatal development. An important question, then, is whether pharmacological interventions in the neonate can result in a significant drug-nutrient interaction that alters the maturing biochemical and physiological processes involved in drug absorption/disposition processes and nutrient homeostasis.

In a proof-of-concept investigation, we used the conditionally essential nutrient, L-carnitine, and the β-lactam antibiotic, cefepime, both known to be substrates for L-carnitine transporters expressed at epithelial barriers and cellular membranes. Maintenance of L-carnitine levels is important for normal mitochondrial and epithelial/endothelial functions that are crucial for normal neonatal growth and development [20,21]. During postnatal life, the major pathways involved in L-carnitine homeostasis undergo significant ontogenesis [20,22,23]. The organic cation/carnitine transporter 2 (Octn2) is of particular importance in the maintenance of L-carnitine homeostasis [24,25]. The substrate specificity of Octns also includes a variety of therapeutically used cationic drugs such as the β-lactam antibiotics [26,27]. Both *in vitro* and *in vivo* research have identified significant competitive interactions between such drugs and L-carnitine [26,28]. 

In acknowledgement of the paucity of information regarding drug-nutrient interactions during early postnatal life we used the known L-carnitine and cefepime competitive transporter interaction as the underlying premise to a significant drug-nutrient interaction in the neonate to identify possible alterations in the ontogeny of L-carnitine homeostasis pathways. We also examined whether the consequences of this putative drug-nutrient transporter interaction depend on the timing and duration of drug exposure relative to postnatal maturation. The overall purpose of our study was to provide the first experimental evidence of alterations in the ontogeny of nutrient homeostasis pathways as a result of a significant drug-nutrient transporter interaction during postnatal development. Our study design did not allow assessments of whether such changes were transient or permanent and this would be a focus for further investigations. 

## 2. Materials and Methods

### 2.1. Animals, Diet, and Chemicals

Female Sprague-Dawley rats at gestation day 16 were obtained from Charles River Canada (St. Constant, PQ) and were housed singly in a temperature and humidity controlled facility (22 ºC ± 2 ºC) on a 12-hour light: dark cycle (0700–1900 h). All rats had free access to food and water throughout the study and were allowed a 7-day acclimatization period. All procedures were conducted in accordance with the Canadian Council of Animal Care guidelines for the care and use of laboratory animals and were approved by the Animal Care and Supply Committee of the University of Saskatchewan. Throughout the acclimatization period and during the study, rats had available *ad libitum* a rat diet (Prolab® RMH 3000, Purina, Inc., Richmond, IN) meeting the nutritional requirements for lactating Sprague-Dawley rats.

Cefepime was purchased from the Royal University Hospital at the University of Saskatchewan (Saskatoon, SK). RNeasy Midi kits were obtained from Qiagen Inc. (Mississauga, ON). The Advanced Protein Assay kit was obtained from Fluka (Sigma-Aldrich, MO), QuantiTect SYBR Green RT-PCR kit was purchased from Applied Biosystem (Foster City, CA). L-carnitine and other chemicals not otherwise specified were obtained from Sigma-Aldrich (St. Louis, MO).

### 2.2. Chronic Cefepime Administration in Rat Pups

Cefepime (5 mg) or saline (control) was subcutaneously administered to rat pups (n = 6 dams per dosing schedule) twice daily using the following schedules: 1) postnatal day 1 to day 4; 2) postnatal day 1 to day 8; 3) postnatal day 1 to day 20; 4) postnatal day 8 to day 11; and 5) postnatal day 8 to day 20. These dosing schedules considered short-term and long-term cefepime treatment and developmental maturation of L-carnitine biosynthesis pathways in rat pups (postnatal day 8) [20,29]. Total body weights were recorded daily. At the end of each dosing schedule rat pups were humanely euthanized and blood, heart, intestine, kidney and liver were collected. All tissues were snap frozen in liquid nitrogen and stored at -80 ºC. Blood and tissues were pooled from 5 pups from each mother such that the dam was considered the experimental unit.

### 2.3. Free L-Carnitine Analysis in Serum and Heart

Serum and heart free L-carnitine was quantified by HPLC-UV with pre-column derivatization according to Feng *et al.* [30]. Briefly, the pooled heart samples were homogenized with phosphate buffer (50 mM, pH 7.4) in a ratio of 50 mg tissue:250 µL buffer. The homogenate was centrifuged at 2500 × g for 10 min at 4 ºC. The supernatant or serum (20 μL) sample was precipitated using 9:1 acetonitrile:methanol in a 1:9 v/v. A 300 mg mixture of Na_2_HPO_4_ and Ag_2_O (9:1 wt/wt) and 300 mg of KH_2_PO_4_ were added followed by 1 h vortex-mixing. Derivatization reagent (40 mg/mL ρ-bromophenacyl bromide with 50 μL 40% tetrabutylammonium hydroxide solution) was added into the organic extract. The reaction mixture was incubated at 60 ºC for 2 h followed by centrifugation at 12,000 × g for 15 min. L-Carnitine was analyzed using a Hewlett Packard 1050 HPLC system with Diode Array Detector, Quaternary Pump and Autosampler. Samples (10 μL) were injected onto a CN (cyano) column (HyperClone 5 µm, 250 × 4.6 mm, Phenomenex, Torrance, CA) with detection wavelength set at 260 nm. The mobile phase (90% acetonitrile/10% mM citrate phosphate buffer, pH 3) was delivered at a flow rate of 1 mL/min. The standard curve range was linear (r^2^ > 0.99) between 2.5-40 μmol/L. Intra- and interassay accuracy and precision ranged from 6%-14%.

### 2.4. Total mRNA Isolation and Quantitative RT-PCR Analysis

Total mRNA was extracted from different tissues using RNeasy Midi Kits according to manufacturer instructions. RNA purity and quantity was determined spectrophotometrically by measurement at 260 nm and the OD260/OD280 ratio, respectively, using a Synergy HT Multi-Mode Microplate Reader (Biotek instrument, USA). Total RNA was stored at -80 ºC until analysis. Gene sequences were obtained from the National Center for Biotechnology Information GeneBank and specific primers were designed using Primer3 software (http://primer3.sourceforge.net/) (Table 1). Quantitative RT-PCR (QRT-PCR) analysis was carried out using a QuantiTect SYBR Green RT-PCR kit and an Applied Biosystems 7300 Real-Time PCR system (Carlsbad, CA). The QRT-PCR protocol was carried out according to manufacturer’s instructions. The protocol consisted of reverse transcription (1 cycle at 48 ºC, 30 min), PCR initial activation step (1 cycle at 95 ºC, 15 min), three-step thermal-cycling (40 cycles; denaturing at 94 ºC for 15 seconds, annealing at 60 ºC for 30 seconds, and primer extension at 60 ºC for 30 seconds), and a melt curve analysis from 65 ºC-95 ºC at 0.5 ºC/second.

**Table 1:** Primer sequences for quantitative RT-PCR of L-carnitine homeostasis pathway mechanisms.

Gene	Accession Number	Primers	
		Forward	Reverse
β-actin	NM_031144	agcgtggctacagcttcacc	tgccacaggattccataccc
Octn1	NM_022270	catggctgtgcagactgg	gcaccatgtagccgatgg
Octn2	NM_019269	ggcgcaaccacagtatcc	ggggctttccagtcatcc
Octn3	NM_019723	gacaccgtgaacctgagc	ccatccaggcagttctcc
Cpt1b	NM_013200	cagccatgccaccaagatc	aagggccgcacagaatcc
Cpt2	NM_012930	gctccgaggcgtttctca	tggccgttgccagatagc
Bbh	NM_022629	acgatggggcagagtcc	ctggcctcctgagaaaagc
Tmlh	NM_133387	aatgtccctcccactcagg	tcggtatggcgatctaggg

Octn – organic cation/carnitine transporter 1; Cpt – carnitine palmitoyltransferase 1b; Bbh – γ-butyrobetaine hydroxylase; Tmlh – trimethyllysine hydroxylase.

### 2.5. Validation of the 2^-ΔΔCT^ Method

Real-time PCR assays were initially optimized to give PCR efficiency between 1.9-2.1 (as determined by a 3-point standard curving using serial dilutions of control RNA with a slope range of -2.9 to -3.5) and a single melt-peak corresponding to the appropriate PCR product as verified by 2% agarose gel electrophoresis. The reactions were further optimized for usage of the 2^-ΔΔCT^ method using β-actin as an internal standard. The amplification efficiency of each target and β-actin was determined by constructing a standard curve from crossing point values (C_T_) and RNA concentration. The target genes and β-actin were then amplified using the same diluted samples. The ΔC_T_ were calculated (*i.e.* the difference between the target gene C_T_ and β-actin C_T_). Only primers giving PCR amplification close to 100% and the relative efficiencies between the target and β-actin that were approximately equal were used in our experiment (*i.e.* the slope from log RNA concentration *versus* ΔC_T_ were < 0.1). Fold differences in mRNA expression between control and treated samples were then calculated.

### 2.6. Liver γ-Butyrobetaine Hydroxylase (Bbh) Enzyme Activity

Liver tissue was homogenized in homogenization buffer consisting of 300 mM sucrose, 1 mM EGTA, and 50 mM Tris, pH 7.5 using 1:4 mass to volume ratio. The homogenate was then centrifuged at 13,000 × g for 30 min at 4 ºC. The supernatant was collected and centrifuged at 100,000 × g for 1 h at 4 ºC. 300 μL of the supernatant representing the cytosolic fraction containing Bbh was then transferred into a dialysis tubing cellulose membrane (molecular weight cutoff of 12,000Da, D9777, Sigma, USA), and submerged in 5 L dialysis buffer (75 mM KCl, 0.1mM DTT and 0.5 mM EDTA in sodium phosphate buffer, pH 7.4) overnight in 4 °C. The dialyzed sample (20 μL) was tested for L-carnitine residue and the remaining dialysate was stored at -80 ºC for Bbh testing. The optimal protein concentration, substrate concentration and reaction time to give linear product formation was determined for each age group. For determination of Bbh activity in rat pup livers, 1 mL reaction buffer consisting of 0.2 mM γ-butyrobetaine, 20 mM potassium chloride, 3 mM 2-oxoglutarate, 10 mM sodium ascorbate, 0.4 mg/mL catalase in 20 mM potassium phosphate buffer pH 7.0 was prepared. The reaction was initiated by adding 2 µL ferrous ammonium sulfate (FAS) (final concentration was 0.25 mM) and 20 µL enzyme into 78 µL reaction buffer and incubated for 25 min at 37 ºC using a Boekel/Grant Orbital and Reciprocating Water Bath (Model ORS200, ExpotechUSA, Houston, TX). The reaction was then terminated by adding 10× volume of acetonitrile:methanol (9:1). The mixture was centrifuged at 13,000 × g for 2 min and the supernatant was used for L-carnitine analysis by HPLC.

### 2.7. Heart Carnitine Palmitoyltransferase (Cpt) Enzyme Activities

Heart Cpt enzyme activities were measured using the spectrophotometric method described by Bieber *et al.* [31]. Briefly, frozen heart tissue was homogenized in 10% (wt/v) homogenization buffer (20 mM HEPES, 140 mM KCl, 10 mM EDTA and 5 mM MgCl_2_, pH 7.4) supplemented with 3 mg nagarse using a Polytron homogenizer (Brinkmann Instruments, Rexdale, Canada). The homogenate was then centrifuged at 500 × g for 10 min at 4 ºC. The supernatant was collected in new tubes and centrifuged at 9000 × g for 35 min at 4 ºC. The pellet was then washed with the homogenization buffer without nagarse and centrifuged at 9000 × g for 35 min at 4 ºC. The washed pellet was resuspended in 200 μL isolation buffer without nagarse. Protein concentrations were measured using the Advanced Protein Assay kit (Bio-Rad Laboratories Canada, Mississauga, ON) with bovine serum albumin as standards. The optimal protein concentration and reaction time to give linear product formation were initially determined. To determine total Cpt activity 20 µg protein was assayed in 200 µL ml reaction buffer containing 20 mM HEPES, 1 mM EGTA, 220 mM sucrose, 40 mM KCl, 0.1 mM 5,5’-dithio-bis (2-nitrobenzoic acid) (DTNB), 1.3 mg/mL BSA, and 40 μM palmitoyl-CoA, pH 7.4. The reactions were initiated by adding 1 mM L-carnitine and read at 412 nm after 5 min incubation at 37 ºC using Synergy HT Multi-Mode Microplate Reader (Biotek instrument, USA). Cpt2 activity was determined using the same reaction conditions as total Cpt except 10 µL Cpt1 inhibitor, malonyl-CoA, was added into 200 μL of the reaction mixtures to obtain a final concentration of 10 μM. Cpt1 activity was calculated by subtracting the Cpt2 activity from the total Cpt activity. The Cpt activity was calculated as amount of CoASH released per min per mg protein, which is based on the 5-thio-2-nitrobenzoate formation from CoASH-DNTB reaction. The extinction coefficient for 5-thio-2-nitrobenzoate quantification was 13.6 mM/cm [32,33].

### 2.8. Heart High Energy Phosphate Substrate Determination

The heart high energy phosphate substrate levels including creatine (Cr), creatine phosphate (CrP), ATP, ADP and AMP were measured with HPLC-UV method as described by Olkowski *et al*. [34]. The heart samples were homogenized in 0.7 M ice cold perchloric acid (MW 100.46) with a final concentration 100 mg/mL. The homogenate was centrifuged at 12,000 rpm for 5 min. The supernatant was collected and neutralized with 2 M potassium hydroxide to bring pH near to 7.0. The supernatant was then filtered through 0.45 µm filter (Nonsterile Syringe Filter Nylon, Chromatographic Specialties Inc. Brockville, Ontario, Canada) and 10 µL was injected into a 3 μ Luna C-18 (Phenomenex, Torrance, CA) column using step gradient flow conditions. The mobile phase components used included 20 mM potassium phosphate buffer (pH 7.0) and 100% methanol was delivered at a flow rate of 1 mL/min in the sequence of 100% phosphate buffer from 0-6.5 min, 100% methanol from 6.5-12.5 min followed by 100% phosphate buffer from 12.5 to 25 min for column re-equilibration, which was sufficient to achieve stable baseline conditions. The elution profile of high energy phosphate substrates including CrP, Cr, ATP, ADP and AMP were monitored at 210 nm. The standard curve range was from 6.25-100 µg/mL and the limit of detection was 0.078 µg/mL for ATP and 0.31 µg/mL for ADP and AMP. Intra- and interassay accuracy and precision ranged from 4.2% to 14.5%. 

### 2.9. Histopathology of the Heart

Heart tissue was harvested immediately after the pups were euthanized and preserved in phosphate buffered formalin. Tissue blocks were embedded in paraffin. The tissue was then sectioned into 5 µm thick sections and stained with hematoxylin and eosin. The histological preparations of hearts (six replications per slide) were subjected to detailed microscopic evaluation. For semi-qualitative analysis, the observed lesions were graded according to *a priori* established standards, taking into consideration histopathological features characteristic of cell degeneration such as cytoplasmic eosinophilia, chromatin condensation and nuclear pyknosis, spongy degeneration of the cytoplasm, cytoplasmic vacuolization, and karyorrhexis. The assessment of the degree of myocardial changes was based on an arbitrarily set criteria as follows: 1) ‘No visible lesions’ was assigned (score of 0) when all structures of the myocardial tissue appeared morphologically normal, 2) ‘Mild lesions’ was assigned (score of 1) when changes were predominantly focal or multifocal, but limited to a very small area of myocardium, 3) ‘Moderate lesions’ was assigned (score of 2) when changes were focal, but affecting a large area of myocardium, predominantly prominent multifocal, or smaller scale locally extensive, an 4) ‘Severe lesions’ was assigned (score of 3) when changes were multifocal and affecting larger areas of the ventricular myocardium, or were predominantly locally extensive. In order to ensure unbiased evaluation, two pathologists examined slides with a double blind approach (*i.e.* neither identity of the subjects nor treatments applied were known to the scientists who conducted the evaluation).

### 2.10. Statistical Analysis

All data are reported as mean ± SEM. All experimental data were analyzed using SPSS 13.0 (SPSS Inc., Chicago, IL, USA). The interactions between treatment and duration time for all the selected parameters were assessed by 2 × 2 factorial ANOVA using General Linear Model (GLM) multivariate. Multiple comparisons for all the parameters at different ages were analyzed using one way ANOVA with Fisher's least significant difference (LSD) as post hoc test. The effects of cefepime on rat pups in each treatment duration period were analyzed by one way ANOVA or unpaired t-test as appropriate. In addition, Pearson's correlation coefficients were computed to quantify the association between all the processes involved in maintaining L-carnitine homeostasis (serum free L-carnitine levels) during development. Data used for this purpose include serum L-carnitine levels, mRNA expression level of kidney Octn2, intestinal Octns, Tmlh mRNA expression, and Bbh mRNA expression and activity in control rats from postnatal day 4, day 8, day 11 and day 20.

## 3. Results and Discussion

### 3.1. Body Weight Gain

Cefepime caused no differences in total body weight gain or growth pattern regardless of dosing schedule (P > 0.05) (data not shown). However, cefepime did cause changes in various molecular, biochemical and histological markers depending upon timing and/or duration of cefepime administration relative to postnatal age. 

### 3.2. L-Carnitine Levels

**Figure 1:**
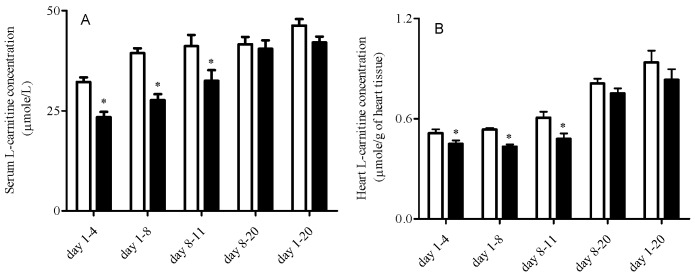
Mean ± SEM free L-carnitine levels in serum **(A)** and heart **(B)** from rat pups treated with saline (white bar) or 5 mg cefepime (black bar) twice a day by subcutaneous injection according to different dosing schedules (n = 6). Means were compared by one way ANOVA with LSD as post hoc analysis; *α = 0.05.

Cefepime caused significant decreases in serum L-carnitine levels when rat pups were treated from day 1-4, day 1-8 and day 8-11 (P < 0.05) with no differences in the other dosing schedule groups (P > 0.05) (Figure 1A). Cefepime caused significant decreases in heart L-carnitine levels when rat pups were treated from day 1-8 and day 8-11 (P < 0.05) with reduced L-carnitine levels from day 1-4 (P = 0.07) and no differences in the other groups (P > 0.05) (Figure 1B). 

*3.3. mRNA Expression Levels of L-Carnitine Transporters*

Cefepime treatment caused no difference in heart Octn2 expression (P > 0.05) (Table 2). Kidney Octn2 mRNA expression levels were significantly upregulated in treatment groups from day 1-4, day 8-20 and day1-20 (P < 0.05) with no difference in the other groups (P > 0.05) (Table 2). The mRNA expression of Octn2 was significantly upregulated in the intestine after cefepime treatment from day 8-11 (P < 0.05) with no differences in other treatment groups (Table 2). Cefepime treatment caused a significant increase in intestinal Octn1 mRNA expression levels in treatment groups day 8-11 and day 1-20 (P < 0.05) (Table 2). Intestinal Octn3 mRNA expression levels were also enhanced by cefepime exposure in treatment groups day 1-8 and day 1-20 (Table 2).

**Table 2:** Mean ± SEM fold differences relative to control in mRNA expression level of L-carnitine transporters (Octns) in kidney, heart and intestine, liver Bbh and Tmlh and heart Cpts from rat pups treated with 5 mg cefepime or saline (control) twice daily by subcutaneous injection according to different dosing schedules (n = 6).

	Day 1-4	Day 1-8	Day 8-11	Day 8-20	Day 1-20
Kidney Octn2	1.27 ± 0.10^*^	1.17 ± 0.15	0.98 ± 0.14	2.24 ± 0.37^*^	1.24 ± 0.05^*^
Intestinal Octn1	0.99 ± 0.09	0.90 ± 0.09	1.89 ± 0.33^*^	0.84 ± 0.08	1.80 ± 0.16^*^
Intestinal Octn2	1.18 ± 0.28	1.06 ± 0.16	1.22 ± 0.10^*^	1.09 ± 0.17	0.76 ± 0.21
Intestinal Octn3	1.39 ± 0.50	1.84 ± 0.26^*^	1.02 ± 0.17	2.21 ± 0.89	1.51 ± 0.12^*^
Heart Octn2	0.99 ± 0.03	0.88 ± 0.13	0.95 ± 0.14	1.09 ± 0.12	0.92 ± 0.10
Liver Bbh	1.15 ± 0.56	0.90 ± 0.26	0.89 ± 0.20	0.73 ± 0.22	0.90 ± 0.33
Liver Tmlh	0.82 ± 0.18	1.58 ± 0.28^*^	1.12 ± 0.12	1.28 ± 0.09	1.18 ± 0.21
Heart Cpt1b	1.02 ± 0.17	1.03 ± 0.15	1.34 ± 0.22	1.00 ± 0.13	0.83 ± 0.09
Heart Cpt2	1.19 ± 0.12	0.97 ± 0.07	1.32 ± 0.16	1.15 ± 0.18	1.10 ± 0.09

Expression was normalized to ß-actin and fold difference was determined using the comparative C_T_ method for relative quantitation. Means between control and cefepime treatment were compared using unpaired t-test; *α = 0.05.

### 3.4. Liver Bbh and Tmlh and Heart Cpt Enzyme Expression and Activity

**Figure 2:**
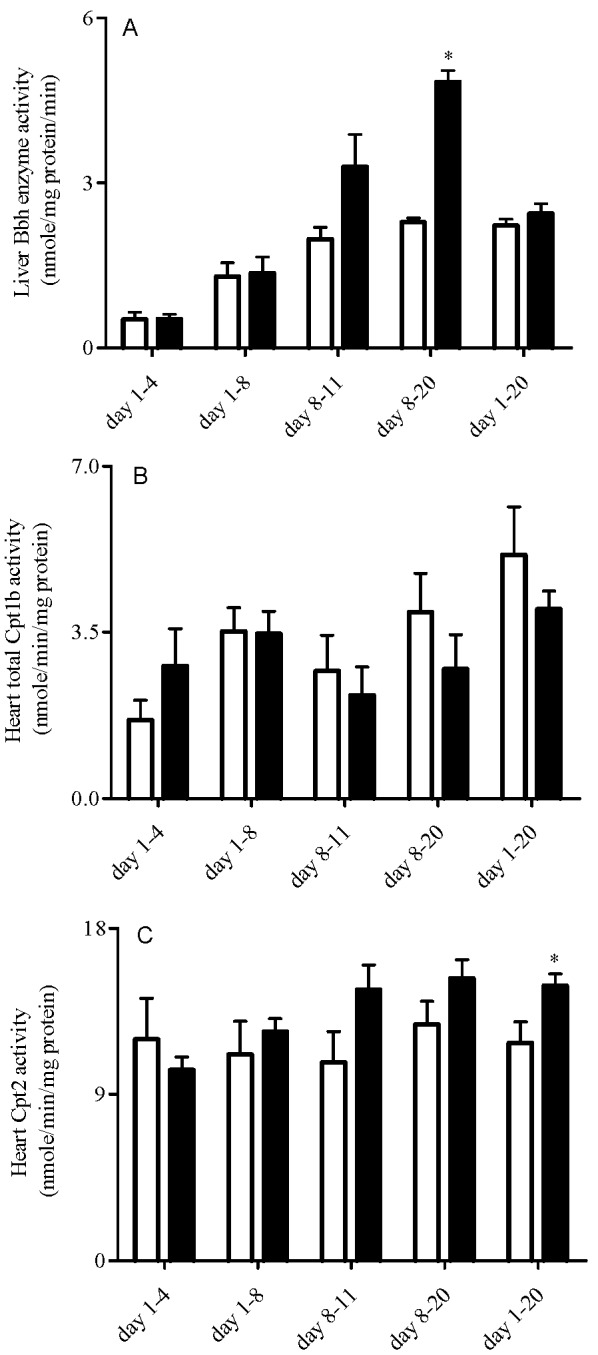
Mean ± SEM activities of liver Bbh **(A)**, heart Cpt1 **(B)** and heart Cpt2 **(C)** in rat pups treated with saline (white bar) or 5 mg cefepime (black bar) by twice daily subcutaneous injection according to different dosing schedules (n = 6). Means were compared using one way ANOVA with LSD as post hoc analysis; *α = 0.05.

Cefepime caused no changes in Bbh mRNA expression levels in all treatment groups (P > 0.05), but enhanced Tmlh mRNA levels in postnatal day 1-8 group (Table 2). Bbh enzyme activities were significantly upregulated in rat pups treated from day 8-20 (P < 0.05). In addition, Bbh enzyme activities were higher in rat pups treated from day 8-11 though the changes were not statistically significant (P = 0.06) (Figure 2A). Cefepime caused significant increases in heart Cpt1b mRNA expression levels (Table 2) and Cpt2 activities in rat pups treated from day 1-20 (P < 0.05) with no effect on other treatment groups (Figure 2B and Figure 2C). 

### 3.5. Heart High Energy Phosphate Substrate Levels

**Figure 3:**
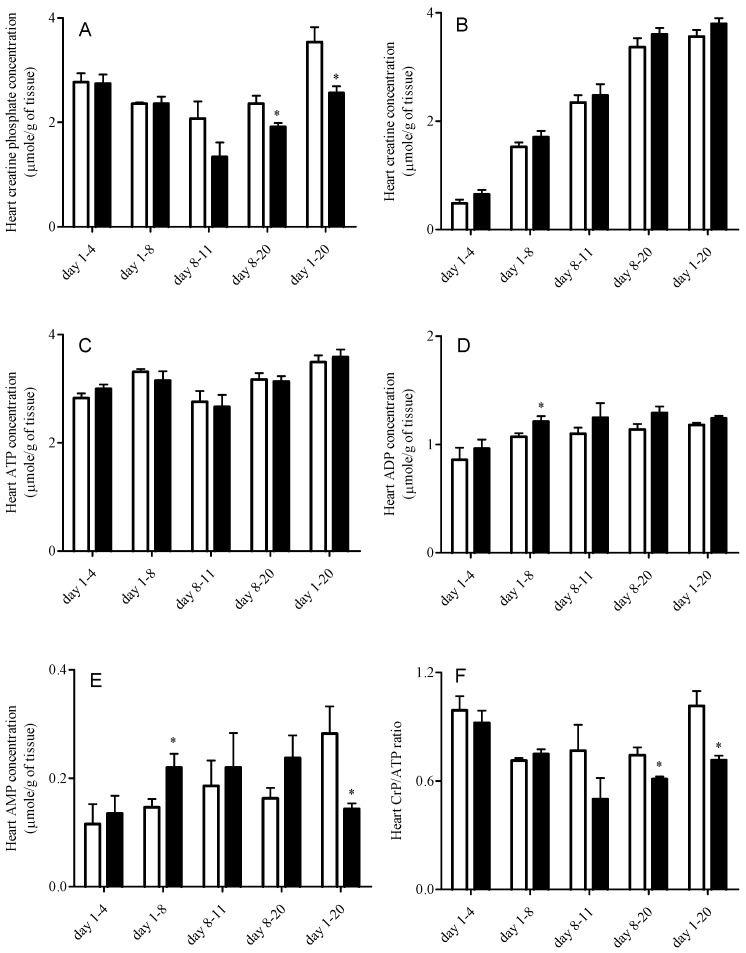
Heart high energy phosphate substrate profiles in rat pups treated with saline (white bar) or 5 mg cefepime (dark grey bar) twice daily by subcutaneous injection according to different dosing schedules (n = 6). **(A)** creatine phosphate; **(B)** creatine; **(C)** ATP; **(D)** ADP; **(E)** AMP; **(F)** CrP/ATP ratio. Means were compared with one way ANOVA with LSD as post hoc analysis; *α = 0.05.

Figure 3 presents the heart high energy phosphate substrate profiles measured in rat pups. Creatine phosphate concentration in rat pup hearts was reduced by cefepime treatment from postnatal day 8-20 and day 1-20 (P < 0.05). ADP levels were significantly increased in treatment group postnatal day 1-8 (P < 0.05) with some increase in day 8-20 (P = 0.08) and day 1-20 (P = 0.08). In addition, AMP levels were significantly increased in postnatal day 1-8 treatment group but decreased in postnatal day 1-20 treatment group (P < 0.05). The ratio between CrP and ATP (CrP/ATP ratio) was significantly reduced in postnatal day 8-20 and day 1-20 treatment groups (P < 0.05).

### 3.6. Histological Analysis

**Figure 4:**
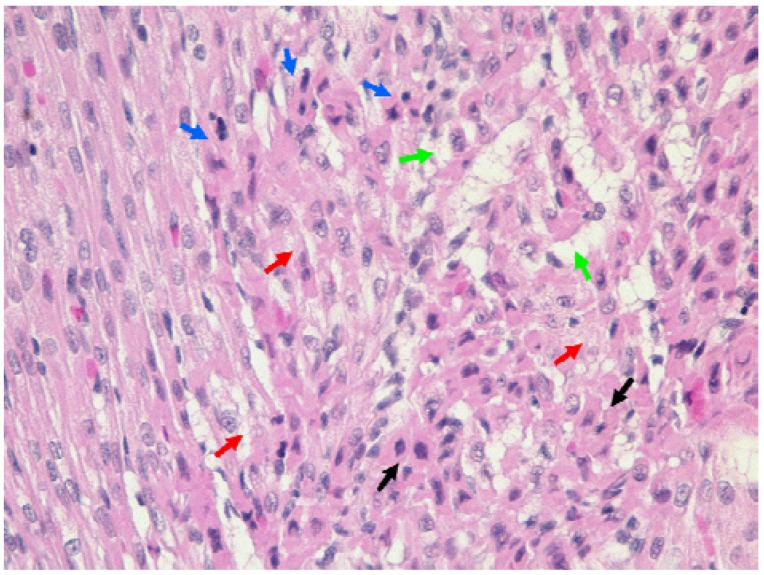
Representative histopathological features of the mural ventricular myocardium of rat pup heart (the specimen is from rat pup treated with cefepime from day 1-8). The significance of the arrows and arrowheads is described in the results section of the text (original magnification 400×).

The heart specimens from both control and treated animals showed some pathological changes indicative of myocardial tissue degeneration. However, lesions were more severe in myocardium of rat pups treated with cefepime from day 1-4, day 1-8 and day 8-11 with similar, but less severe, lesions in rat pups treated from day 8-20 and day 1-20 (Table 3). Representative examples of severe changes are shown in Figure 4. Noteworthy are early degenerative changes marked by cytoplasmic eosinophilia and nuclear pyknosis (black arrows). The affected cardiomyocytes had a distinct dull dark pink appearance, and had lost their typical striation pattern. Some cardiac cells showed aggregation of the nuclei. A more advanced pathological process in the cardiomyocytes was evidenced by karyorrhexis (blue arrows). Many cells showed spongy degeneration of the cytoplasm, and in some instances cytoplasmic vacuolization was prominent (red arrows). The myocardial syncytium was punctuated by clear spaces between the myofibers and interstitial edema was present in some areas. Cardiomyocyte dropout was evident in more severely affected areas (green arrows) (Figure 4). 

**Table 3:** Mean ± SEM Scores of cardiac lesions in rat pups treated with saline (Control) or 5 mg cefepime (Treated) by twice daily subcutaneous injection according to different dosing schedules (n = 6).

	Day 1-4	Day 1-8	Day 8-11	Day 8-20	Day 1-20
Control	1.67 ± 0.49	2.17 ± 0.31	1.17 ± 0.40	1.33 ± 0.42	1.00 ± 0.52
Treated	2.50 ± 0.34	2.83 ± 0.17	2.17 ± 0.31	1.17 ± 0.40	0.33 ± 0.33
P value	0.196	0.086	0.076	0.781	0.304

Means between control and cefepime treatment were compared using unpaired t-test; *α = 0.05. The histopathological score scheme used in this study was: No visible lesion – 0; Mild lesion – 1; Moderate lesion – 2; and Severe lesion – 3.

### 3.7. Correlation Analysis

Cefepime treatment changed the maturation patterns of intestinal Octn2 and Octn3 mRNA expression levels, concentrations of CrP, ATP and AMP, and Cpt1 activities (P > 0.05) (Table 4). Significant interactions between treatment and duration times on the serum L-carnitine levels, liver Bbh activities and heart AMP levels was indicated on statistical analysis (P < 0.05).

**Table 4:** Pearson’s correlation coefficients between postnatal ages and all parameters assessed in rat pups treated with saline (Control) or 5 mg cefepime (Treated) by twice daily subcutaneous injection (rat pups were from day 4, day 8, day 11 (n = 6 for each age) and day 20 (n = 12).

Parameters	Control Animals		Treated Animals	
	Pearson Correlation Coefficient	P value	Pearson Correlation Coefficient	P value
Serum L-carnitine	0.642	0.000	0.861	P < 0.0001
Heart L-carnitine	0.840	P < 0.0001	0.846	P < 0.0001
Kidney Octn2 mRNA	0.369	0.045	0.665	P < 0.0001
Heart Octn2 mRNA	0.827	P < 0.0001	0.879	P < 0.0001
Intestinal Octn1 mRNA	0.305	0.101	0.370	0.044
Intestinal Octn2 mRNA	-0.465	0.010	0.083	0.663
Intestinal Octn3 mRNA	0.803	P < 0.0001	0.098	0.608
Liver Bbh activity	0.417	0.022	0.707	P < 0.0001
Liver Bbh mRNA	0.034	0.857	0.387	0.035
Liver Tmlh mRNA	0.047	0.807	-0.037	0.848
CrP	0.248	0.186	-0.168	0.375
Cr	0.956	P < 0.0001	0.956	P < 0.0001
ATP	0.421	0.021	0.358	0.052
ADP	0.533	0.002	0.419	0.021
AMP	0.415	0.023	0.093	0.627
Cpt1 activity	0.564	0.001	0.268	0.152
Cpt2 activity	0.086	0.653	0.603	0.000
Heart Cpt1b mRNA	0.692	P < 0.0001	0.382	0.037
Heart Cpt2 mRNA	0.838	P < 0.0001	0.901	P < 0.0001

### 3.8. Discussion

Neonates can be exposed to drugs therapeutically and through secondary exposure (e.g., via breast milk). When drug exposures are intentional, dosage regimens consider the developmental changes in drug pharmacokinetic processes in pediatric patients with dosage adjustments made according to maturation of enzymes and transporters involved in drug absorption and disposition [35]. Some of these pharmacokinetic processes that undergo developmental maturation are also utilized by various nutrients in the body. In particular, a variety of transporters have been identified as having substrate specificities that include both nutrients and therapeutically used drugs. Thus, co-administration of a drug may cause a functional interaction with the transporters responsible for nutrient absorption and/or disposition, and other pharmacokinetic processes shared between a drug and nutrient. Knowing that the body may adapt strategies to maintain optimal nutrient homeostasis in the presence of a nutritional imbalance [36,37], such an interaction has the potential to change the ontogeny of nutrient homeostasis pathways in developing neonates. If the interaction occurs during a susceptible window of vulnerability the interaction may result in metabolic programming of the nutrient homeostasis pathway(s), the consequences of which may impact an individual’s risk for development of disease [38]. 

The present study used the conditionally essential nutrient, L-carnitine, and a known inhibitor of L-carnitine transport, cefepime, to examine possible alterations in the ontogeny of L-carnitine homeostasis pathways. This study demonstrated that cefepime administration altered L-carnitine homeostasis pathways in neonatal rats. Of particular note was the dependence upon duration and timing of exposure relative to postnatal development on the qualitative and quantitative changes observed with cefepime exposure. Serum and heart free L-carnitine levels were reduced in groups which started treatment early and lasted shorter (day 1-4, day 1-8 and day 8-11). In contrast, L-carnitine levels remained within developmentally normal levels when rat neonates were treated with cefepime until weaning (day 1-20, and day 8-20) (Figure 1). Since L-carnitine is an obligatory nutrient for neonatal development, reduced levels early in postnatal development as a result of the cefepime-L-carnitine interaction may pose a detrimental risk to neonates. The literature has drawn an association between reductions in L-carnitine levels and development of cardiac hypertrophy and cardiomyopathy [39]. In our study, histopathological examinations revealed severe degenerative changes in ventricular myocardium in treatment groups with reduced L-carnitine levels. Although minor histopathological changes were identified in groups with normal free L-carnitine blood levels and rats pups treated with saline, these changes were likely attributed to the stress of twice daily injections. The severe degenerative changes in the younger neonates with reduced L-carnitine levels may eventually lead to compromised heart function and a predisposition to cardiovascular disease in later life.

When alterations in nutrient availability occur, the body may have a variety of adaptive responses to maintain nutrient homeostasis. L-Carnitine homeostasis is maintained primarily through endogenous biosynthesis in the liver, exogenous dietary sources, and renal reabsorption of filtered L-carnitine [40]. However, newborns depend almost exclusively on exogenous sources of L-carnitine [41] until the biosynthesis and renal uptake systems for L-carnitine mature with advancing age [24]. The postnatal maturation of different key systems involved in L-carnitine homeostasis allows for a potential “window(s) of susceptibility”. Transporters mediate both the gastrointestinal absorption and active reabsorption of L-carnitine from the urinary filtrate and such transporters account for more than 75% of body L-carnitine [24]. In the adult, renal L-carnitine transport systems are known to rapidly adapt to dietary L-carnitine levels through modifications in L-carnitine reabsorption efficiency at the kidneys [42]. Our results suggest that neonates also have the capacity to adjust their renal L-carnitine reabsorption by changing Octn2 expression levels, the principle transporter involved in the active reabsorption of L-carnitine from the urinary filtrate [43]. Renal Octn2 mRNA expression was highly upregualted in the rat pups demonstrating normal L-carnitine levels despite cefepime treatment (day 8-20 and day 1-20) (Table 2). Since Octn2 accounts for more than 95% of L-carnitine reabsorption from the filtrate [43], the increase in Octn2 expression may significantly contribute to the maintenance of L-carnitine levels in these two groups despite competitive interactions with cefepime at renal Octn2. 

Duration of treatment also seemed to influence the extent to which renal Octn2 expression increased with cefepime treatment. Limited to no significant changes in expression was observed with short-term cefepime treatment. Although renal Octn2 mRNA expression was also increased in the postnatal day 1-4 treatment group, the enhanced expression was not adequate to assure sufficient reabsorption of L-carnitine in the presence of a competitive interaction with cefepime since serum L-carnitine levels were reduced. 

In addition to renal Octn2, cefepime also influenced intestinal Octn expression, but the qualitative and quantitative changes varied depending on the treatment schedules. Alterations in intestinal Octn expression may be the result of an adaptive response to altered L-carnitine levels following a competitive interaction with cefepime. Intestinal transporters are known to undergo adaptive regulation according to nutrient status [44,45,46]. Although a competitive interaction at gastrointestinal L-carnitine transporters in rat pups is unlikely following subcutaneous administration, a competitive interaction at renal reabsorption mechanisms may lead to reduced systemic L-carnitine levels and a need to alter intestinal L-carnitine transporters in response to altered L-carnitine status. Interestingly, cefepime administration did not influence heart Octn2 expression in any of the postnatal groups evaluated. These data suggest that regulation of heart Octn2 expression is refractory to the interaction between cefepime and L-carnitine unlike Octn2 expression in the kidney and intestine. 

Endogenous biosynthesis of L-carnitine, particularly by the liver, accounts for 25% of L-carnitine in the adult. However, immaturity of hepatic γ-butyrobetaine hydroxylase (Bbh) activity at birth limits the ability of neonates to biosynthesize L-carnitine. In the rat, Bbh activity reaches adult capacity by postnatal day 8 [20]. Interestingly, our study demonstrated that hepatic Bbh activity was affected only when cefepime treatment was instituted after Bbh maturation (*i.e.* day 8), while Tmlh mRNA expression was increased in the postnatal day 1-8 treatment group. This increase in Tmlh and the enhancements in Bbh activity in treatment groups postnatal day 8-20 (P < 0.05) and day 8-11 (P = 0.06) may be an adaptive response to compensate for the loss of L-carnitine in the body via competitive interactions at renal Octn2. Cefepime exposure did not alter Bbh maturation when treatment was initiated in early postnatal life and had no affect on Tmlh expression with initiation of treatment after maturation of Bbh expression. These observations may suggest that the maturation of hepatic Bbh activity is genetically programmed and may not be influenced by either internal (*i.e.* L-carnitine concentration) or external factors (*i.e.* cefepime exposure). This phenomenon has been noted for other nutrient homeostasis pathways, such as in the case of age related changes in monosaccharide uptake rate, which undergoes genetically programmed maturation and is only slightly influenced by dietary manipulations [47]. 

L-Carnitine plays an obligate role in energy production as it facilitates the transport of long chain fatty acids across the mitochondrial membrane making them available for β-oxidation [24,48]. Tissues with low L-carnitine levels often have low fatty acid oxidation rates [49]. Despite reductions in serum and heart L-carnitine levels in some treatment groups in our study, Cpt activity and heart Octn2 expression was not altered and cefepime exposure did not influence ATP levels in rat pup hearts. However, alterations in other high energy phosphate substrates including ADP, AMP and CrP were observed depending on the treatment schedules. Unfortunately, the specific interrelationships between the changes in these high energy phosphate substrates are difficult to rationalize in our study. However, we did identify significant decreases in CrP and the CrP/ATP ratios in treatment groups with normal L-carnitine levels (Day 8-20 and day 1-20). These finding are significant because both the loss of creatine phosphate and the reduction in the ratio of CrP/ATP has been associated with several pathological conditions in animals [50]. Moreover, the reduction of CrP/ATP ratios has been used as a predictor of mortality for patients with dilated cardiomyopathy [51], which is a typical symptom of L-carnitine deficiency [52]. The decreased CrP levels and CrP/ATP ratios may be early signs of heart dysfunction in these cefepime treated animals despite the fact that serum and heart L-carnitine levels were maintained at the normal range. 

For many physiological and biochemical processes considerable maturation of these processes continues throughout neonatal development. In the rat, many components involved in the L-carnitine homeostasis pathway undergo significant ontogeny in the period from birth to weaning (manuscript in preparation). In this study, treatment of rat pups with cefepime, a known inhibitor of L-carnitine transport, altered various L-carnitine homeostasis mechanisms during postnatal development. The qualitative and quantitative changes in these pathways depended upon when during postnatal development the neonate was exposed to the drug, as well as to the duration of the exposure. However, despite compensatory changes in renal and intestinal L-carnitine transport systems, rat pups treated at postnatal day 1-4, 1-8 and 8-11 could not maintain serum and heart L-carnitine concentrations at levels associated with their untreated counterparts. As well, these pups showed more severe histopathological changes in the heart relative to their untreated counterparts and to older treated pups. These data suggest that although young neonates have the ability to mount an adaptive response to exogenous factors that influence nutrient status, this adaptive response may not be sufficient depending upon the magnitude of the environmental insult. Our data also generally showed that neonates exposed to cefepime later in postnatal development or throughout the postnatal period seemed to mount an adaptive response that was sufficient to maintain serum and tissue L-carnitine levels and prevent pathological changes in the heart. 

## 4. Conclusion

Our study, which was premised on the known competitive interaction between cefepime and L-carnitine at L-carnitine transporter systems, demonstrated that cefepime administration to neonatal rat pups caused significant alterations in the ontogenesis of several mechanisms involved in the L-carnitine homeostasis pathway. These alterations likely represented adaptive responses to cefepime-induced alteration in L-carnitine status. The qualitative and quantitative changes in these L-carnitine homeostasis pathways seemed to depend upon duration and timing of exposure relative to postnatal maturation, but whether these changes remain permanent are unknown. Future studies are planned to assess the potential for metabolic programming of L-carnitine homeostasis mechanisms following a cefepime-L-carnitine interaction and the long-term consequences of this interaction on the risk for disease later in life. Nonetheless, our findings could have major implications with respect to drug treatment of paediatric patients, particularly young neonates, and highlights a potential need for nutritional interventions during drug therapy in this population. 
